# Evaluation of esthetic outcome of delayed implants with de-epithelialized free gingival graft in thin gingival phenotype with or without immediate temporization: a randomized clinical trial

**DOI:** 10.1186/s40729-023-00468-0

**Published:** 2023-02-13

**Authors:** Maha Fawzy, Manal Hosny, Hani El-Nahass

**Affiliations:** Department of Oral Medicine and Periodontology, Faculty of Dentistry, Cairo University, 11 El-Saraya St. – Manial, Cairo, Egypt

**Keywords:** Dental implants, Immediate loading, Thin phenotype, De-epithelialized Free Gingival Graft

## Abstract

**Aim:**

The current study investigates the effect of immediate temporization on the pink esthetics of delayed implants in patients with thin gingival phenotype in combination with a De-epithelialized Free Gingival Graft in the maxillary premolar area.

**Methodology:**

The study population was randomly assigned into two groups. The two groups were treated with delayed implants with simultaneous placement of a de-epithelialized free gingiva graft. The test group was immediately temporized while the control group had no temporization. The pink esthetic score was assessed as the primary outcome. Additional secondary outcomes were assessed such as the keratinized tissue width and the soft tissue thickness.

**Results:**

Twenty implants were placed in the current study, split into 10 implants per group. The results showed that the Pink Esthetic Score of the IT group was 11.88 ± (1.13) and 11.33 ± (1.25) for the CTG group, which showed no statistical difference between the groups after 1 year of follow-up. There was also no significant difference between the two groups at 12 months regarding the keratinized tissue width and the soft tissue thickness.

**Conclusions:**

Immediate and delayed temporizations have no effect on the Pink Esthetics of the delayed implants; however, immediate temporization allowed earlier provisional crown delivery. Soft tissue augmentation of the thin gingival phenotype improved esthetics for both groups.

*Trial registration* Name of the registry: clinicaltrials.gov; trial registration number: NCT03792425. Date of registration: January 3, 2019. URL of trial registry record: https://clinicaltrials.gov/ct2/show/NCT03792425?term=NCT03792425&draw=2&rank=1

**Graphical Abstract:**

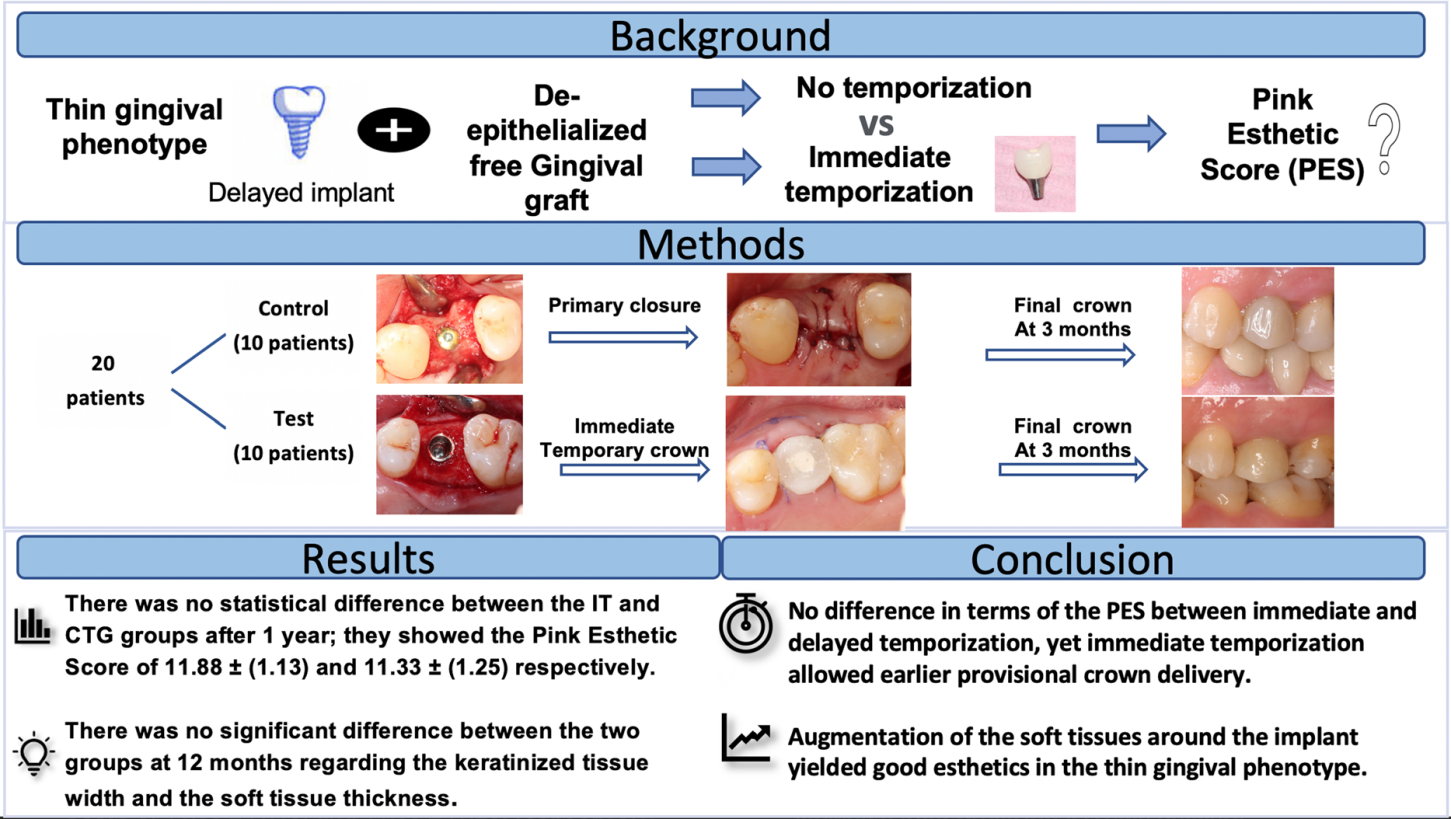

## Main text

Implant dentistry has become an integral part of modern dentistry, and its success is no longer determined merely by osseointegration. Several factors have a pivotal role in the success of implants; among them, is the stability of the peri-implant soft tissues that maintain function and esthetics [1, 2].

Long-term peri-implant soft tissue stability is affected by the peri-implant phenotype [3–5]. A gingival thickness of ≥ 2 mm is defined as a thick phenotype capable of withstanding mastication’s daily wear and tear [6]. In contrast, a gingival thickness of < 1.5 mm is defined as a thin phenotype predisposing the tissues to attachment loss [7]. Patients with a thin peri-implant mucosal phenotype are at a higher risk of suffering marginal recessions or the appearance of the metallic shadow of the implant [8, 9]. Therefore, augmentation of the peri-implant mucosa in such cases is recommended to ensure an adequate quantity and quality of the peri-implant soft tissues [10, 11].

Soft tissue augmentation procedures are designed to increase the thickness of the buccal peri-implant mucosa, resist recession and crestal bone resorption, and provide superior esthetics and long-term health [12]. Besides improving the phenotype, augmenting the soft tissues is also recommended from an esthetic point of view to compensate for the volume loss after tooth extraction [13]. Various materials have been used to perform soft tissue grafting around dental implants. Nevertheless, the connective tissue graft remains the gold standard providing the most predictable results [14, 15], specifically the de-epithelialized free gingival grafts (FGG). This technique provides a firmer and more uniform subepithelial connective tissue graft [16–19]. Beyond augmenting the peri-implant mucosa, immediate temporization has been increasingly utilized in implant placement procedures. This treatment modality serves to mold the peri-implant mucosa and improves the esthetic outcome by optimizing the restoration’s emergence profile. Minor defects can also be treated with proper molding of the peri-implant tissues during the initial healing phase to obtain adequate tissues before final restoration fabrication [20]. Immediate temporization with simultaneous application of the subepithelial connective tissue graft with immediate implants has been widely employed for esthetics and function [13, 14, 19, 21–25].

The current study aims to evaluate the peri-implant soft tissue esthetics following immediate temporization with delayed implant placement and connective tissue grafting in patients with a thin gingival phenotype compared to conventional loading in the maxillary premolar zone.

The outcomes assessed are the Pink Esthetic Score (PES), gingival thickness, and keratinized tissue width.

## Materials and methods

### Study design and registration

The current study was designed as a randomized, controlled, parallel-group clinical trial, following the CONSORT guidelines. The Ethical Committee of the Faculty of Dentistry, Cairo University (January 2019) approved the study protocol, and it was registered in the Clinical Trials Registry (clinicalTrials.gov) NCT03792425.

### Sample size determination

A sample size of 20 implants was calculated for the current study, with 10 implants in each group. This number was based on a study by Weisner et al. [26], where the reported pink esthetic scores were 11.32 ± 1.63. Based on a null hypothesis and a power of 0.85, 8 subjects in each group were found sufficient to reject the hypothesis. This number was increased to 10 in each group to compensate for the losses during follow-up. The sample size was calculated by the G*Power program (University of Dusseldorf, Dusseldorf, Germany).

### Eligibility criteria

All patients included in the study were selected from the outpatient clinic of the Department of Oral Medicine and Periodontology—at Cairo University. Patients included in the study had to have a missing maxillary premolar surrounded by sound neighboring teeth, a thin gingival phenotype ≤ 1.5 mm, and adequate ridge dimensions to receive a regular implant. Medically compromised patients, smokers, and pregnant females were excluded from the study.

A cone-beam computed tomography (CBCT) was taken to determine eligibility for the study to evaluate the Bucco-palatal bone dimensions, including width, height, and density. These measurements were used to determine the appropriate implant length and diameter.

### Randomization

Once the patients were enrolled in the study and informed consent was signed, all patients were randomized using computer-generated randomization (www.randomizer.org) in a 1:1 ratio. The allocation sequence was generated by (S.H) who was not involved in the study. The randomized sequence was placed in opaque, sealed, and sequentially numbered envelopes and broken on the day of the surgery. Patients were randomly allocated into either the control group (delayed implant placement with connective tissue graft only) CTG or the intervention group (delayed implant placement with connective tissue graft and immediate temporization) ITG.

### Pre-operative phase

A thorough pre-operative assessment of all study subjects was carried out to include medical history, dental history and intraoral clinical examination.

Pre-operative measurements were taken, including the soft tissue phenotype and the width of keratinized gingiva. The gingival phenotype was examined by transgingival piercing using a needle and an endodontic stopper [60]. After giving local infiltration anesthesia [26], the measurements were taken at 3 different points; 2 mm, 4 mm, and 6 mm from the crest of the bone [27].

The width of keratinized gingiva was measured at the mid-buccal area by a periodontal probe^1^ from the gingival margin to the mucogingival junction.

An impression was taken for all eligible patients to create a study cast, which was used to fabricate a vacuum stent to be used postoperatively to protect the palate’s de-epithelialized connective tissue donor site.

### Surgical and prosthetic procedures

Following the administration of local anesthesia,^2^ a crestal incision was created using a 15c scalpel [2], and a full-thickness mucoperiosteal flap was raised with minimal reflection to expose the crest of the bone [28]. A buccal pouch was then created by extending the reflection in an apical direction to accommodate the connective tissue graft [29]. Sequential drilling was then done following the manufacturer’s^3^ instructions to allow implant placement. The implant was leveled to the alveolar bone crest, and primary stability was achieved [30].

Once the implant was in place, a free gingival graft was harvested and de-epithelialized extra-orally [31] to produce a connective tissue graft of about 1.5 mm in thickness [26]. The CT graft was tucked into the pouch and sutured to the buccal flap with a resorbable suture^4^ [32]. The graft dimensions were determined based on the dimensions of the edentulous site. Gel foam was placed in the donor site and sutured in place then the stent was fixed in place [26]*.*

**Fig. 1:**
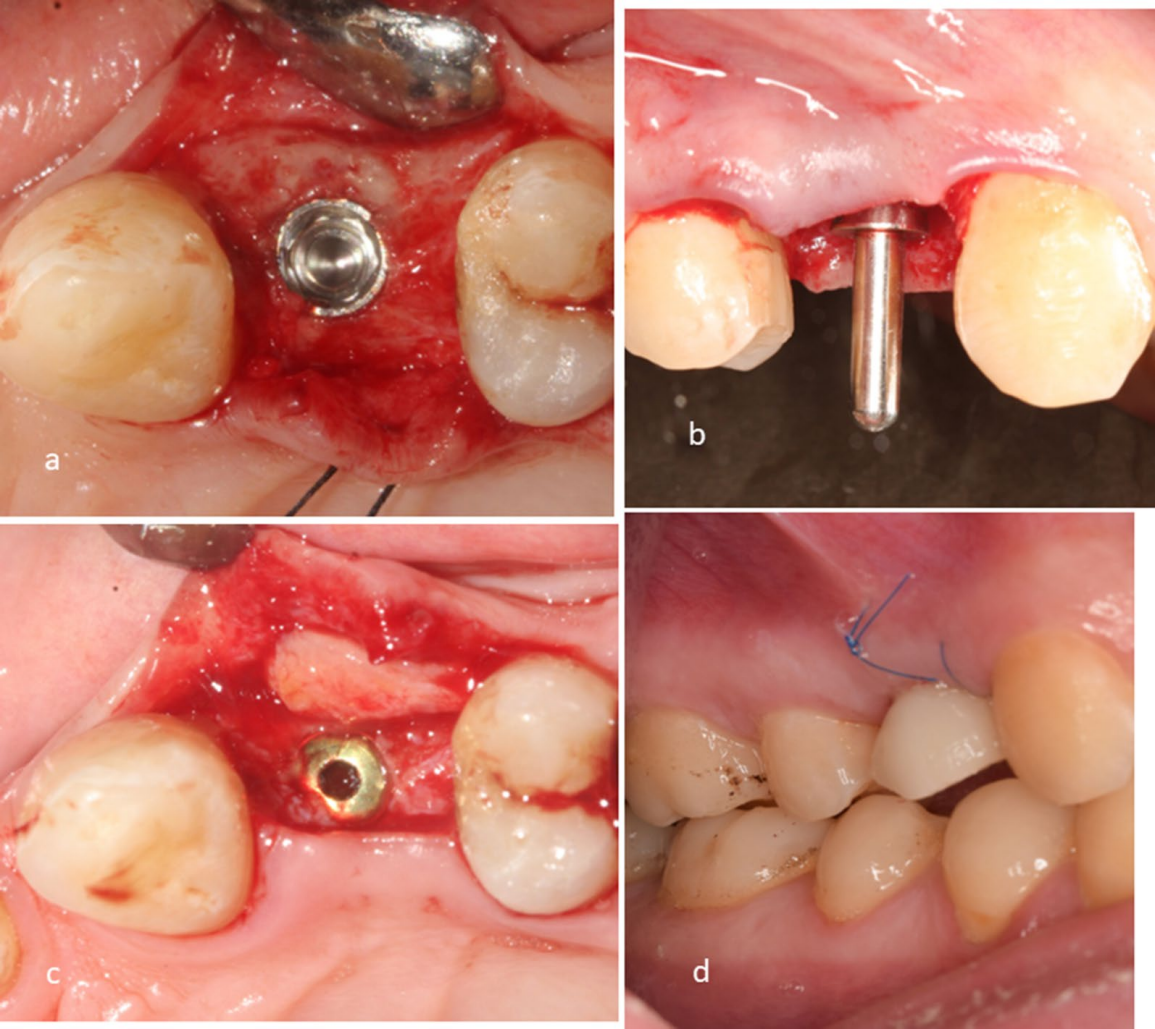
Test group **A**: Implant Placement, **B**: Paralleling Pin, **C**: Connective tissue graft placement in the buccal pouch, **D**: PMMA temporary crown delivered 48 hrs after implant placement and put out of occlusion

In the IT group (Fig. 1), an open tray impression was taken after implant placement but before graft fixation to avoid damaging the graft. A healing collar was placed over the implant and the surgical site was closed with interrupted sutures. A PMMA temporary crown was fabricated on a temporary abutment and delivered to the patient within 48 h of the surgery. The temporary crown was placed out of occlusion for 3 months [2]. Immediate non-functional loading was only done if adequate primary stability ≥ 25 Ncm was obtained.

**Fig. 2:**
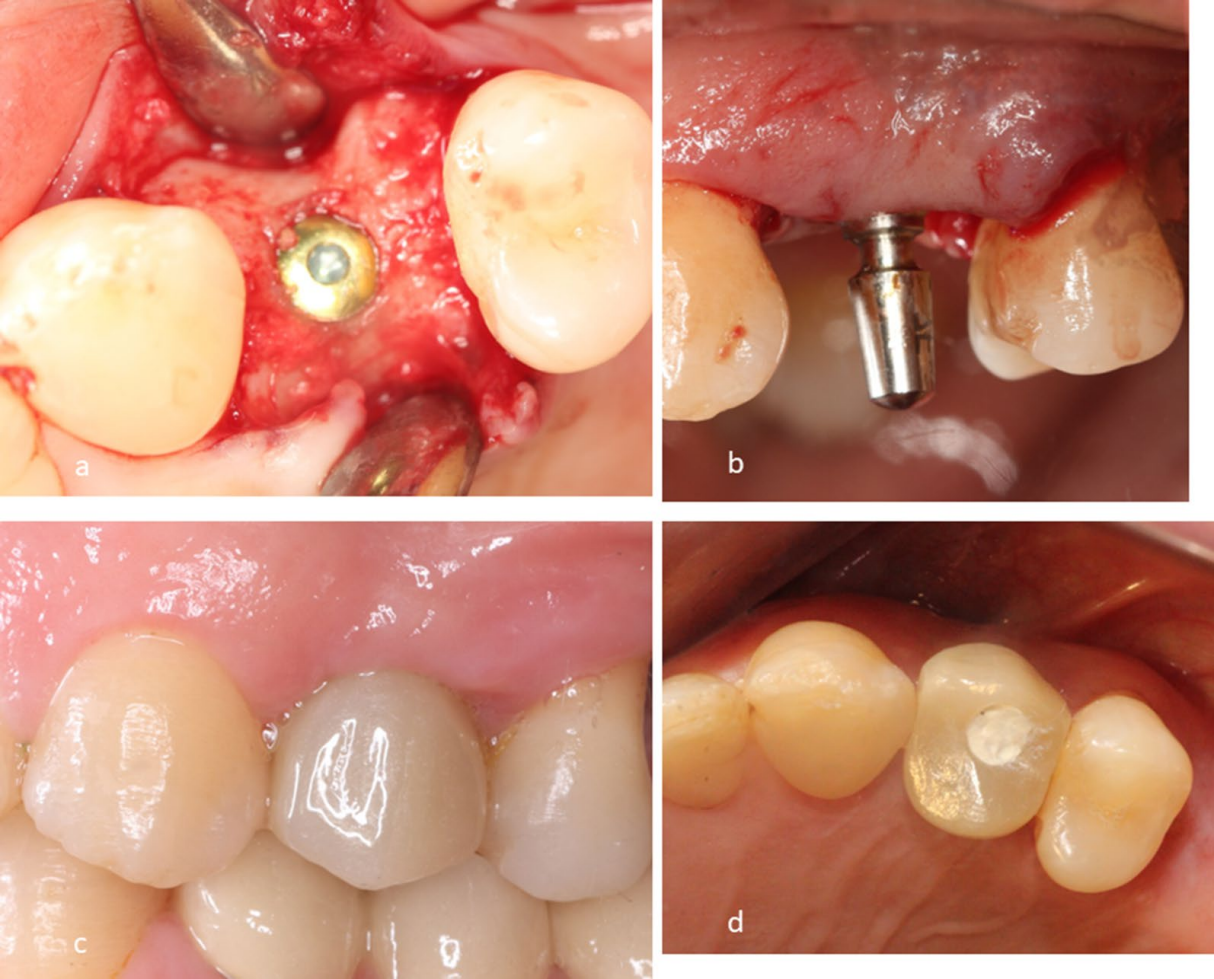
Control group **A**: Implant Placement, **B**: Paralleling Pin, **C**: Final crown delivery, **D**: Occlusal view of the final crown

In the CTG group (Fig. 2), the flap was sutured with interrupted sutures [26] and left to heal. Implant exposure was done 3 months later by creating a T incision [33], and then a healing collar was placed for about 2 weeks to allow soft tissue molding and maturation.

Postoperative medications included anti-inflammatory drugs NSAIDs; Ibuprofen 600 mg three times daily for 3 days, and antiseptic mouth rinse (0.2% Chlorhexidine oral rinse) prescribed two times a day for 2 weeks [2]. Patients were instructed to refrain from hard brushing and trauma to the surgical site for 1 week.

All final impressions were taken indirectly, using an open tray technique, and the final CAD-CAM zirconia crowns were fabricated for all the study subjects [26]. Both groups received their final zirconium crown 3 months after implant placement.

Follow-up visits were done at 3, 6, 9, and 12 months after implantation. Digital pictures were taken for the PES evaluation, and gingival thickness and keratinized tissue width measurements were recorded at the follow-up visits.

#### Calibration

Blinding of the patients and the operator was not possible, but the outcome assessors and the statistician were blinded.

#### Pink Esthetic Score (PES)

Examiners applied the PES index used by Fürhauser [34] to assess the soft tissue around the implant. The PES was evaluated 4 times: 3 months post-implant insertion, after 6 months, 9 months, and at 12 months postoperatively. Every crown was photographed with a digital camera^5^ with the reference tooth completely visible to ensure comparability.

The esthetic evaluations of the soft tissue PES were evaluated by two assessors (M.T) and (R.W) who were not part of the treatment procedures. The assessors were calibrated before the study on 20 single implant cases, then after 1 week, asked to reexamine the same cases in a different order. The frontal color pictures were re-scored twice with an interval of 1 week. Furthermore, the intra-examiner reproducibility was evaluated.

#### Gingival thickness

Gingival thickness was measured by piercing the gingiva along the long axis of the implant at 3 different points at 2 mm, 4 mm, and 6 mm below the center of the crest of the ridge into the mucosa until it contacts the cortical bone [26, 27, 60]. Postoperatively, readings were taken at 4-time points: 3, 6, 9, and 12 months.

#### Keratinized tissue width

It was measured by a periodontal probe (see Footnote 1) from the gingival margin to the mucogingival junction in the mid-buccal area. Postoperatively, readings were taken at 4-time points: 3, 6, 9, and 12 months.

### Statistical analysis

Numerical data are reported as mean and standard deviation, and nominal data are reported as frequency. Nominal data were analyzed using Fisher’s exact test. Numerical data were explored for normality using the Kolmogorov–Smirnov test and the Shapiro–Wilk test. In the case of normally distributed numerical variables, both groups were compared with an independent t-test. In contrast, for non-normally distributed variables, the Mann–Whitney *U* test was a more appropriate choice. For intragroup analysis in normally distributed data, repeated measure ANOVA was utilized. In case it reported a statistical significance, post hoc multiple comparisons were made with Bonferroni adjustments. For non-normally distributed data, intragroup comparisons were performed using the Friedman test. In case it was statistically significant, the post-hoc Wilcoxon Signed Ranks Test was applied for pairwise comparisons. All tests were two-tailed, and P-value less than or equal to 0.05 was considered statistically significant. Data were analyzed using SPSS advanced statistics (Statistical Package for Social Sciences version 26, BM Inc., Chicago, IL).

## Results

### Demographic data (Table 1)

**Table 1:** Demographic Data

	IT	CTG	Intergroup *p*-value
Age (mean, SD)	34.00 (4.57)	36.11 (5.49)	0.406
Gender	10 (females)	10 (females)	
Distribution (first premolar/second premolar)	3/6	4/5	0.637
Implant diameter (3.5 mm/4.00 mm)	(6/3)	(7/2)	0.60

*NC* not computable

In the current study, all participants were females, with an average age range of 34 years in the IT group and 36.11 years in the CTG group. The osseointegrated implants were distributed among the sites as follows 3 implants in the first premolar region and 6 implants in the second premolar region in the intervention group; 6 of them were of diameter 3.5 mm and 3 implants were 4 mm, whereas in the control group; 4 implants were placed in the first premolar region and 5 in the second premolar region; 7 of them were of 3.5 mm diameter while 2 of them were 4 mm with no significance in the distribution between the two groups *p* = 0.637.

### Implant survival

Within the twenty patients enrolled in the study, randomly assigned as ten participants in each group, no participants were lost to follow-up. Two implants out of 20 had early failure; one was in the intervention group (survival rate of 90%), and one was in the control group (survival rate of 90%).

### Pink Esthetic score (Table 2)

**Table 2:** Pink Esthetic Score

	IT Mean (SD)	CTG Mean (SD)	Intergroup *p*-value
PES at 3 months	9.50 (0.53)^a,b^	9.39 (0.65)^a,b,c^	0.708
PES at 6 months	10.75 (0.96)^c,d^	10.61 (0.99)^a^	0.774
PES at 9 months	11.69 (1.13)^a,c^	11.17 (1.03)^b^	0.336
PES at 12 months	11.88 (1.13)^b,d^	11.33 (1.25)^c^	0.365
Intragroup *p*-value	< 0.001*	0.001*	

Intragroup time points with the same letter indicate statistical significance

*Statistically significant

Since two blinded examiners assessed the PES, intra-examiner reproducibility was evaluated resulting in a reliability of 0.826 (95% CI 0.728–0.891) while the correlation coefficient between the 2 examiners reached (ICC) = 0.85.

PES intergroup assessment was calculated using the independent *t*-test, while intragroup measurements were assessed using repeated measure ANOVA showing no significant difference between the two groups.

### PES at 12 months (Table 3)

**Table 3:** PES at 12 months

	IT (SD)	CTG (SD)	*P*-value
Mesial papilla	1.69 (0.37)	1.44 (0.30)	0.138
Distal papilla	1.56 (0.32)	1.50 (0.35)	0.703
Gingival level	1.88 (0.35)	1.67 (0.25)	0.068 NS
Gingival contour	1.63 (0.26)	1.50 (0.50)	0.602 NS
Gingival texture	1.69 (0.26)	1.67 (0.35)	1.00 NS
Gingival color	1.69 (0.37)	1.78 (0.36)	0.547 NS
Alveolar process	1.75 (0.38)	1.78 (0.36)	0.865 NS

*NS* non-significant, *p*-value > 0.05

On reviewing the individual elements of the Pink Esthetic Score for each group.

Mann–Whitney *U* test.

## Discussion

Esthetics is now considered a mandatory factor in assessing implant success and must be considered a priority and a goal side to side with osseointegration [1, 14, 32, 35]. Our study aimed to assess whether immediate temporization with a de-epithelialized subepithelial connective tissue graft in delayed implant placement would enhance the pink esthetics in patients with a thin gingival biotype. The current study aimed to close the knowledge gap highlighted in a review article by Atieh [19], where it was noted that the effect of the gingival phenotype and width of the keratinized tissue were not clearly discussed in the literature. To the best of our knowledge, this is the first study comparing immediate temporization to conventional loading with de-epithelialized free gingival graft in patients with a thin gingival phenotype in delayed implants [44–46].

Immediate temporization allows the shortening of the healing period and early optimization of the esthetics. It also has a pivotal effect on the shaping of the peri-implant soft tissues, papilla fill, and producing the proper emergence profile for single-tooth implant restorations [2, 24, 41–43]. Combining the effect of immediate temporization with simultaneous soft tissue grafting is advantageous to the healing process compared to delayed temporization that require a second surgery for implant exposure. Performing multiple surgical procedures in a single site is associated with shrinkage as the wound edges get drawn together [39, 40]. This shrinkage occurs as a proportion of the fibroblasts begin to mature into a phenotype that resembles the smooth muscle cells [38] leading to pronounced shrinkage. Even when considering soft tissue grafting, it is known that subepithelial connective tissue grafts are associated with shrinkage (25–45%) that occurs within the first month but could be detected up to 1 year [36, 37].

Patients with thin gingival phenotypes are at a higher risk of esthetic complications in the long term. The appearance of a greyish shadow of the implant, midfacial peri-implant mucosal recession, incomplete papilla fill, and decreased soft tissue stability are among those esthetic complications [8, 47–50], so the gingival thickness is considered a key factor in achieving the ideal esthetic outcome. Crestal bone stability has been linked to the thicker phenotype [48, 51, 52], as the sites with thick gingival phenotypes are less prone to buccal changes compared to thin gingival biotypes. Adequate amount of tissues is needed to curb the crestal bone loss and achieve the biological width of the peri-implant mucosa or the supra crestal tissue height [48, 51, 52]. Therefore, augmentation is beneficial in patients with a thin gingival phenotype receiving an implant in the esthetic zone to ensure better thick peri-implant mucosal tissues.

The present study’s primary outcome was the pink esthetic score as it could objectively assess esthetics [14, 34, 53]. Two different blinded examiners (M.T, R.W.) recorded the readings due to the subjective nature of the PES outcome, and an average between the two scores was calculated (Table 2). The CTG group yielded a score of 11.33 ± 1.25 at 1-year follow-up. This agreed with findings reported by [26], who reported a PES of 11.32 ± 1.63 after 1 year. Bruyckere et al. [54], demonstrated in an RCT utilizing connective tissue graft to re-establish buccal convexity, the PES of the CTG graft group after a 1-year follow-up was 10.48 ± 2.25.

There was no statistical difference between the IT and CTG groups after 1 year as they showed 11.88 ± 1.13 and 11.33 ± 1.25, respectively. The IT group did not result in better PES. Nevertheless, it allowed the patient to have a provisional crown at an earlier stage, therefore, saving the treatment time. The esthetics of single delayed implant placement in a thin phenotype represents a challenge to clinicians. The current study shows that subepithelial connective tissue grafting with or without temporization could produce excellent esthetics’ results.

The detailed PES did not show a significant difference in the various comparison aspects. Furthermore, both groups showed excellent alveolar process readings, attributed to the connective tissue grafting. This compensates for the volume loss that occurs due to buccal bone resorption after extraction, which is a common defect in delayed implants. Occlusal photos could have given more insights into the alveolar process improvement. It appears that achieving excellent PES is related to soft tissue augmentation rather than the timing of temporization. The changes in peri-implant mucosal soft tissue thickness were evaluated at levels 2, 4, and 6 mm from the mucosal margin of the future restoration (Table 4). Measurements at different levels were performed by [27, 55]. This was adopted in the present study to give a complete picture of changes in the peri-implant mucosa, especially since volumetric changes were not performed. The changes reported in both intervention and control groups (Table 4) showed that maximum gain was achieved within 3 months, and then there was a minor reduction in soft tissue thickness. Similar findings have been reported by [19, 56, 57]**,** and could be attributed to soft tissue grafts undergoing remodeling processes that may start from 1 to 6 months after soft tissue augmentation, according to [13]**.** These results are in line with the results of [55, 56] who reported a comparable increase in mucosal thickness.

**Table 4:** Gingival thickness

	IT Mean (SD)	CTG Mean (SD)	Intergroup *p*-value
At 2 mm level			
Baseline	1.19 (0.26)^a,b,c,d^	1.17 (0.25)^a,b,c,d^	0.862
3 months	2.56 (0.56)^a,e,f^	2.33 (0.56)^a,e,f^	0.250
6 months	2.44 (0.50)^b^	2.22 (0.57)^b^	0.227
9 months	2.31 (0.53)^c,e^	2.06 (0.39)^c,e^	0.234
12 months	2.31 (0.53)^d.f^	2.11 (0.42)^d,f^	0.373
Intra-group *P*-value	< 0.001*	< 0.001*	
At 4 mm level			
Baseline	1.50 (0.00)^a,b,c,d^	1.56 (0.39)^a,b,c,d^	1.00
3 months	2.56 (0.32)^a^	2.39 (0.55)^a^	0.304
6 months	2.50 (0.38)^b^	2.44 (0.30)^b^	0.744
9 months	2.44 (0.32)^c^	2.44 (0.30)^c^	0.955
12 months	2.44 (0.32)^d^	2.44 (0.30)^d^	0.955
Intra-group *P*-value	< 0.001*	< 0.001*	
At 6 mm level			
Baseline	1.50 (0.00)^a,b,c,d^	1.44 (0.17)^a,b,c,d^	0.346
3 months	2.50 (0.38)^a^	2.39 (0.33)^a^	0.525
6 months	2.50 (0.38)^b^	2.39 (0.33)^b^	0.525
9 months	2.44 (0.32)^c^	2.33 (0.25)^c^	0.492
12 months	2.44 (0.32)^d^	2.33 (0.25)^d^	0.492
Intra-group *P*-value	< 0.001*	< 0.001*	

Intragroup time points with the same letter indicate statistical significance

Intergroup: Mann–Whitney test

Intra group: Friedman test, with post-hoc Wilcoxon

*Statistically significant

The results of our CTG group are slightly inferior to those [26], which might be because the study did not consider the gingival phenotype.

In the literature, the studies that evaluated changes in mucosal thickness after concurrent application of C.T graft and immediate temporization were mainly related to immediate implant placement [58]. The palatal positioning of the implants and the concave contoured immediate provisional crowns at the subgingival level creates an internal void between the gingiva and the immediate provisional restoration, which leads to the thickening of the soft tissue thickness in the IT group even without a connective tissue graft [17].

Regarding the keratinized tissue width, there was no significant difference between the two groups, 4.88 ± 0.23 and 4.61 ± 0.42 at 12 months follow-up. Yet, there was a significant intragroup change through time from the baseline values of 4.13 ± 0.23 and 3.94 ± 0.58 for the test and the control groups, respectively (Table 5).

**Table 5:** Keratinized tissue

	IT Mean (SD)	CTG Mean (SD)	Intergroup *p*-value
Baseline	4.13 (0.23)^a,b,c,d^	3.94 (0.58)^a,b,c,d^	0.743
3 months	4.69 (0.26)^a^	4.56 (0.39)^a^	0.487
6 months	4.69 (0.26)^b^	4.56 (0.39)^b^	0.487
9 months	4.81 (0.26)^c^	4.67 (0.43)^c^	0.547
12 months	4.88 (0.23)^d^	4.61 (0.42)^d^	0.155
Intra-group *P*-value	< 0.001*	< 0.001*	

Intragroup time points with the same letter indicate statistical significance

Intergroup: Mann–Whitney test

Intra group: Friedman test, with post-hoc Wilcoxon

*Statistically significant

In the control group, there was a significant increase in KTW from 3.94 ± 0.58 to 4.56 ± 0.39 mm after 6 months. This agrees with [35, 59], which yielded a significant mean KT increase at 1-year follow-up after soft tissue augmentation.

When interpreting the results of the current study, some limitations should be acknowledged. Although favorable results were achieved in this short-term follow-up, larger-scale clinical studies with longer follow-ups are needed to demonstrate good external validity and adequately evaluate the long-term efficacy of such treatment approaches. Furthermore, further research with radiographic tools should be explored to determine labial bone changes, with the peri-implant soft tissue thickness. Finally, all the patients were females, and the missing tooth was located in the premolar area, which could affect the outcomes.

## Conclusion

Augmentation of the soft tissues with connective tissue graft in patients with a thin gingival phenotype produced particularly good esthetics in terms of PES scores with continuous improvement in PES in both groups after permanent restoration up to 12 months. Immediate temporization seemed to improve the overall treatment outcome.

## Recommendations

RCTs with longer follow-up periods, and larger sample sizes are needed to evaluate the benefit of immediate temporization with delayed implant placement and simultaneous connective tissue grafting.

## Data Availability

The datasets used and/or analyzed during the current study are available from the corresponding author upon reasonable request.

## Footnotes

1 UNC—15 Hu friedy.

2 Articaine hydrochloride 4% with 1:100,000 Epinephrine.

3 IS II active, Neobiotech, South Korea.

4 Vicryl Assut suture 5/0.

5 Canon EOS 80 D.
